# Reconnaissance of Oxygenic Denitrifiers in Agriculturally Impacted Soils

**DOI:** 10.1128/msphere.00571-22

**Published:** 2023-04-05

**Authors:** Emily V. Schmitz, Craig L. Just, Keith Schilling, Matthew Streeter, Timothy E. Mattes

**Affiliations:** a Department of Civil and Environmental Engineering, 4105 Seamans Center, The University of Iowa, Iowa City, Iowa, USA; b Iowa Geological Survey, University of Iowa, Iowa City, Iowa, USA; University of Wisconsin-Madison

**Keywords:** oxygenic denitrification, nitric oxide dismutase (*nod*), nitrous oxide, agricultural soil, *Methylomirabilota*, phylogenetic tree

## Abstract

Row crop production in the agricultural Midwest pollutes waterways with nitrate, and exacerbates climate change through increased emissions of nitrous oxide and methane. Oxygenic denitrification processes in agricultural soils mitigate nitrate and nitrous oxide pollution by short-circuiting the canonical pathway to avoid nitrous oxide formation. Furthermore, many oxygenic denitrifiers employ a nitric oxide dismutase (*nod*) to create molecular oxygen that is used by methane monooxygenase to oxidize methane in otherwise anoxic soils. The direct investigation of *nod* genes that could facilitate oxygenic denitrification processes in agricultural sites is limited, with no prior studies investigating *nod* genes at tile drainage sites. Thus, we performed a reconnaissance of *nod* genes at variably saturated surface sites, and within a variably to fully saturated soil core in Iowa to expand the known distribution of oxygenic denitrifiers. We identified new *nod* gene sequences from agricultural soil and freshwater sediments in addition to identifying nitric oxide reductase (qNor) related sequences. Surface and variably saturated core samples displayed a *nod* to 16S rRNA gene relative abundance of 0.004% to 0.1% and fully saturated core samples had relative *nod* gene abundance of 1.2%. The relative abundance of the phylum *Methylomirabilota* increased from 0.6% and 1% in the variably saturated core samples to 3.8% and 5.3% in the fully saturated core samples. The more than 10-fold increase in relative *nod* abundance and almost 9-fold increase in relative *Methylomirabilota* abundance in fully saturated soils suggests that potential oxygenic denitrifiers play a greater nitrogen cycling role under these conditions.

**IMPORTANCE** The direct investigation of *nod* genes in agricultural sites is limited, with no prior studies investigating *nod* genes at tile drains. An improved understanding of *nod* gene diversity and distribution is significant to the field of bioremediation and ecosystem services. The expansion of the *nod* gene database will advance oxygenic denitrification as a potential strategy for sustainable nitrate and nitrous oxide mitigation, specifically for agricultural sites.

## INTRODUCTION

Row crop production in the agricultural Midwest pollutes waterways with nitrate and exacerbates climate change through increased emissions of nitrous oxide (1). In Iowa, the approximately 2 million miles of agricultural drainage infrastructure lowers the water table and decreases soil saturation typically associated with nitrous oxide and nitrate mitigation (2–4). There is a critical need for sustainable nitrate and nitrous oxide mitigation strategies at agricultural sites, especially in Iowa. A promising ecosystem service in addressing nitrate and nitrous oxide pollution is oxygenic denitrification, a recently discovered and unique nitrogen cycling pathway. Oxygenic denitrification bypasses the canonical pathway and avoids nitrous oxide production by employing a nitric oxide dismutase (*nod*) that transforms 2 molecules of nitric oxide into molecular nitrogen and oxygen (5). The intracellularly produced oxygen can be used for aerobic catabolic processes in anoxic environments (5–7). The nitric oxide dismutation step distinguishes oxygenic denitrifiers from traditional denitrifiers and, therefore, can be used as a biomarker to indicate oxygenic denitrification (5, 6).

Some constraints for establishing *nod* genes as effective biomarkers, include unresolved phylogenetic associations of *nod* genes and the limited number of studies directly investigating *nod* genes in the environment. To date, 2 probable *nod* genes (DAMO_2434 and DAMO_2437) have been identified in the fully sequenced genome of the uncultured nitrite-dependent anaerobic methane-oxidizing (n-DAMO) bacterium *Candidatus* Methylomirabilis oxyfera (5). *Candidatus* M. oxyfera is a member of the phylum *Methylomirabilota*, which has been referred to as NC10 (8, 9). A third probable *nod* gene, HdN1_*nod*, was found in the genome of Gammaproteobacterium strain HdN1 (5, 9–11). Nitric oxide dismutation has been suggested in diverse catabolic functions including the oxidation of methane, long chain aliphatic hydrocarbons, benzene, and toluene (11–14). Our expanding understanding of *nod* diversity suggests there are more bacteria with *nod* capacity than the few identified through metagenomics or environmental studies (6, 11, 12).

*Nod* reduces nitric oxide differently from the related quinol-dependent nitric oxide reductase (qNor) due to proposed structural differences essential to functionality and electron supplying mechanisms (5, 12). qNor sequences have signature residues conserved around the quinol-binding site (His328, Glu332, and Phe336) and the catalytic site (His 508, Glu 512, His559, and His 560) that distinguish qNor; however, multiple sequence alignments have illustrated that these residues are not conserved in *nod* sequences (12, 15–17). Additionally, phylogenetic analysis has shown that putative *nod* and qNor sequences form distinct clades (10, 12, 16, 17). However, clusters of unknown qNor-related sequences that phylogenetically lie somewhere in between *nod* and qNor have also been obtained (10, 12, 18). These unknown qNor-related sequences complicate the identification of putative *nod* genes and result in ambiguity of the gene’s inferred function.

Our current understanding of the distribution and potential of oxygenic denitrification is mostly limited to studies investigating n-DAMO bacteria. As oxygenic denitrification enables the n-DAMO process, it is currently inferred that where n-DAMO bacteria have been identified through 16S rRNA genes or other n-DAMO biomarkers, oxygenic denitrification is taking place (5). While analyzing n-DAMO 16S rRNA genes is useful for determining the presence of potential oxygenic denitrifying taxa, it does not allow for strong functional inferences. Furthermore, bacteria with diverse catabolic capabilities other than n-DAMO are known to harbor *nod* genes (11, 17, 18). Consequently, biomarkers that only indicate n-DAMO bacteria are insufficient in capturing the complete spectrum of *nod* genes or oxygenic denitrification occurring at a given site.

*Nod* sequences have been recovered from diverse environments, including a marine oxygen minimum zone, freshwater and aquifer sediments, wetlands, and agricultural soil (5, 11–13, 16–20). However, there is a lack of reported *nod* sequences from agricultural systems. *Nod* gene quantification can be used to represent the potential abundance of oxygenic denitrifiers and provide information about the niche space of these bacteria. However, the quantification of *nod* genes in the environment is even more limited compared to the sequence recovery distribution. The purpose of this study was to investigate the diversity, distribution, and abundance of *nod* genes at several locations in Iowa, including a site containing tile drains. We aim to expand the use of *nod* genes as effective functional biomarkers for oxygenic denitrification in the environment to promote this approach for sustainable nitrate and nitrous oxide mitigation at agricultural sites.

## RESULTS AND DISCUSSION

### Phylogenetic analysis of *nod* genes in Iowa environmental samples.

We hypothesized that *nod* sequences present in agricultural soil would be distinct from sequences obtained from dissimilar habitats, such as freshwater sites. We isolated DNA from 3 freshwater sediment sites, 3 agricultural sites, and 1 soil core (Table 1). To identify and visualize the diversity of *nod* sequences from our sites, we analyzed both phylogenetic relationships and the presence of conserved residual substitutions in inferred *nod* amino acid sequences. We successfully recovered 88 sequences which we organized in a phylogenetic tree to align with reported *nod* clusters. Seven *nod* clusters were previously classified, casually named for the habitat or organisms from where they originated (10, 12). For example, the “aquifer cluster” was first established in a phylogenetic analysis comparing wastewater, reactor, and aquifer sequences (12). Since then, multiple sub-lineages within the aquifer cluster, containing sequences obtained from agricultural soil and freshwater sediment, have been described (12), including a proposed subcluster from an alpine wetland (16). Surprisingly, only 8 *nod* sequences (9.1% of the total sequences in this study), 7 originating from agricultural soil and 1 from freshwater sediments, grouped with the NC10 lineage. This finding suggests that the remainder of the recovered sequences were from phyla other than NC10 (i.e., *Methylomirabilota*).

**TABLE 1:** *Nod* gene and 16S rRNA gene sequence abundance in DNA extracted from various environmental sites.^*a*^

Site description	*Nod* copies/g soil	16S rRNA copies/g soil	Relative abundance	Nitrogen	Reference
Variably saturated environmental samples					
Freshwater riverbank sediments	7.7 × 10^4^	1.9 × 10^9^	0.004%	1.4 mg/l NO_3_-N^*b*^	This study
Freshwater creek sediments	1.4 × 10^6^	1.5 × 10^10^	0.01%		
Freshwater creek Site 2 sediments	3.0 × 10^6^	1.6 × 10^10^	0.016%		
Farm drainage ditch soil	1.9 × 10^6^	7.6 × 10^9^	0.025%		
Farm tile drain soil	4.3 × 10^6^	9.7 × 10^9^	0.045%	1.8 mg/l NO_3_-N^*b*^	
Farm bog soil	4.5 × 10^6^	8.4 × 10^9^	0.054%		
Farm grass waterway soil core	2.5 × 10^4^ – 2.3 × 10^6^	2.5 × 10^7^ – 1.0 × 10^10^	0.004% – 0.1%		
BTEX-impacted aquifer sediments	1.6 × 10^7^	1 × 10^9^	2%	–	(1)
Alpine wetland soils	2.4 × 10^5^	–	–	0.0195 mg/l NO_3_-N	(2)
Mangrove sediments	7.7 × 10^7^	–	–	0.44 mg/kg NO_3_-N	(3)
Hydrocarbon contaminated environment	1.8 × 10^7^	–	–	4.74 mg/kg NO_3_-N	
Agricultural soil	1.5 × 10^7^	1.4 × 10^9^	1.1%	1.8 g/kg TN	(4)
Agricultural soil with fertilizer	1 × 10^7^	9.8 × 10^8^	1.0%	1.9 g/kg TN	
Agricultural soil with fertilizer and straw	1.3 × 10^7^	1.1 × 10^9^	1.2%	2.6 g/kg TN	
Agricultural soil with fertilizer and manure	1.5 × 10^7^	1.2 × 10^9^	1.3%	2.2 g/kg TN	
Saturated environmental samples					
Farm grass waterway soil core	2.7 × 10^5^ – 1. ×10^6^	2.3 × 10^7^ – 9. ×10^7^	1.2%		This study
BTEX-impacted aquifer well sludge	5 × 10^8^	1 × 10^11^	2%	2 mg/l NO_3_	(1)
BTEX-impacted aquifer sludge	8.3 × 10^2^^*c*^	–	–		(5)
Lake sediment (meso-oligotrophic)	3.2 × 10^7^	1 × 10^9^	3.2%	0.2 mg/l TN	(4)
Lake sediment (eutrophic)	1.5 × 10^7^	2 × 10^9^	0.8%	0.5 mg/l TN	
Lake sediment (dystrophic)	2.2 × 10^6^	4 × 10^8^	0.6%	1 mg/l TN	
Lake sediment (dystrophic)	3 × 10^6^	3 × 10^8^	1.0%	1.7 mg/l TN	

a Gene abundances were normalized to soil wet weight. Where available, the amount of nitrogen present (as nitrate-nitrogen [NO_3_-N] or total nitrogen [TN]) is displayed.

b Nitrogen data retrieved from Iowa Water Quality Information System (IWQIS) sensor located in water.

c Primer set nod840F/nod1012R used to obtain this value in the referenced study.

**FIG 1:**
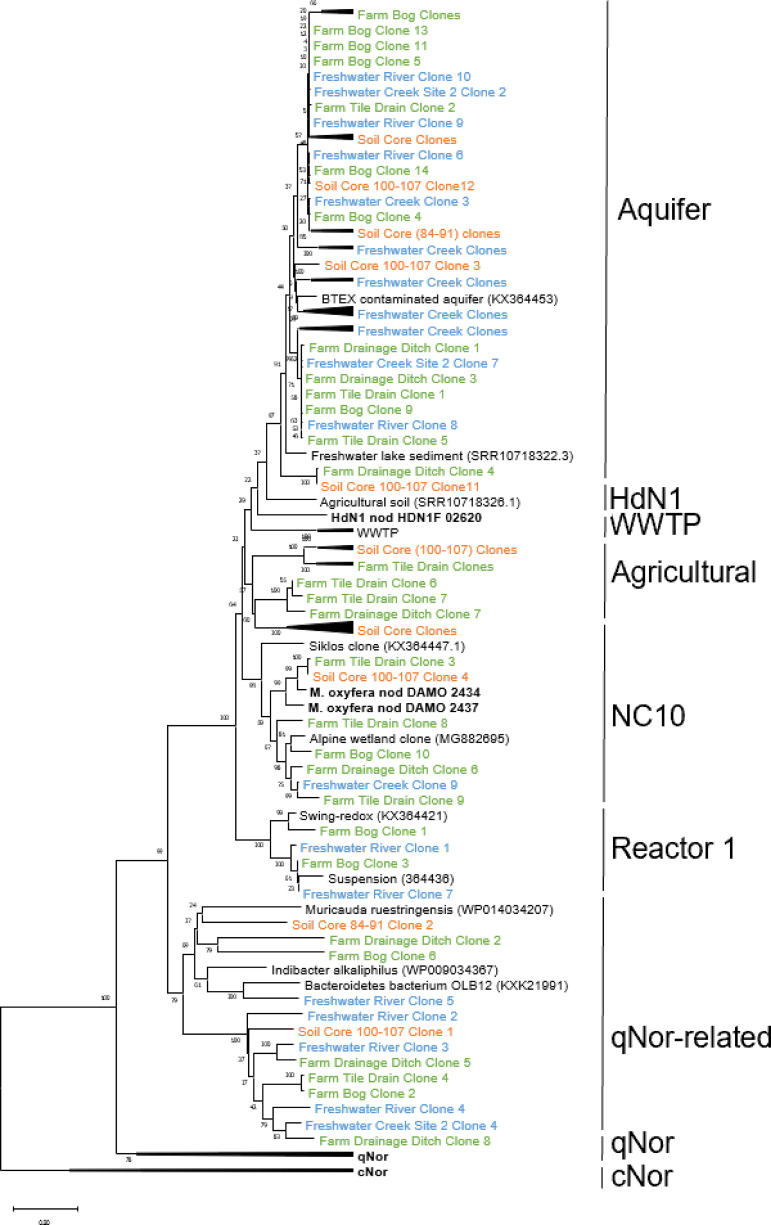
Bootstrapped neighbor-joining amino acid sequence phylogeny of *nod* sequences obtained in this study, along with selected *nod* reference sequences, quinol-nitric oxide reductase (qNor), and cytochrome-nitric oxide reductase (cNor) sequences. *Nod* clones generated in this study are shown in color by their sample location (green, agricultural soil; orange, agricultural soil core; blue, freshwater sediments), and selected reference sequences are shown in black. Our phylogenetic tree illustrates nine clusters designated as follows: aquifer, wastewater treatment plant (WWTP), HdN1, NC10, reactor, qNor-related, qNor, cNor, and one previously unidentified soil cluster (Agricultural). GenBank accession numbers for reference sequences are shown in parentheses, while numbers in parentheses after colored nod sequences indicate the number of sequences in a collapsed group. Bootstrap support (500 replicates) greater than 50% is indicated at the nodes. The scale bar represents 20% amino acid sequence divergence.

Most of the freshwater sediment samples, 22 sequences (71%), were in the more diverse “aquifer cluster”. The remaining freshwater (5 rivers and 2 creek) samples were mostly within the qNor-related lineage and 1 in the NC10 lineage. As expected, none of our agricultural or freshwater sediment *nod* sequences grouped with the “WWTP cluster” or the “HdN1 cluster” (Fig. 1). We also identified a group of 17 *nod* sequences (called “Agricultural”) that were distinct from all previously established *nod* clusters (Fig. 1). These novel sequences were from the farm drainage ditch, farm bog, and agricultural soil cores, understudied habitats that would logically yield sequences not belonging to any of the previously identified *nod*-harboring bacteria. This previously unidentified “agricultural cluster” could be influenced by the presence of elevated nitrogen concentrations compared to the freshwater samples (Table 1). The impact elevated nitrogen concentrations have on oxygenic denitrifiers could be important for understanding the role and impact of oxygenic denitrification in agricultural soils.

Our phylogenetic analysis highlights the role that habitat plays in selecting *nod*-harboring microorganisms as shown by the grouping of sequences obtained from freshwater sediments and agricultural samples. The putative *nod* sequences from the agricultural soils belonged to more clusters than those from the freshwater sediments (Fig. 1), suggesting agricultural soils cultivated more *nod* diversity than freshwater sediments.

**FIG 2:**
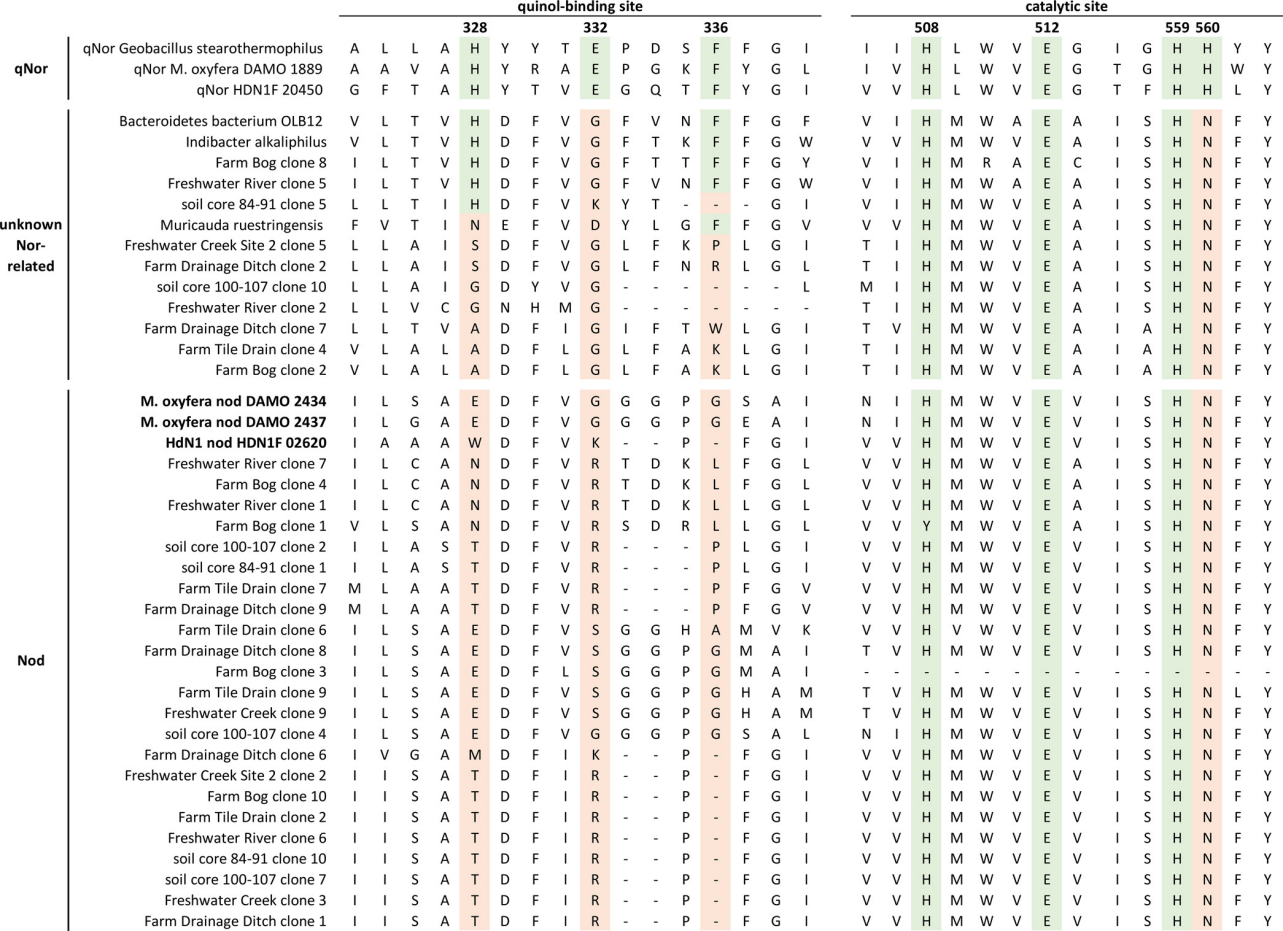
Abridged multiple alignment around the expected quinol-binding and catalytic sites of selected qNor and qNor-related amino acid sequences with selected inferred *nod* amino acid sequences obtained in this study. Conserved quinol-binding and catalytic site residues found in qNor sequences are highlighted in red, whereas substitutions to those conserved residues in putative *nod* sequences are shown in green. The alignment was created in MEGAX with ClustalW using default parameters (31, 32). The unabridged multiple alignment can be found in Fig. S1.

To further confirm the presence of *nod* sequences, we performed multiple alignments of inferred *nod* amino acid sequences from our study with selected *nod* and qNor amino acid reference sequences (Fig. 2, Fig. S2). The analysis targeted the expected locations of the qNor quinol-binding and catalytic sites. Certain residues within the quinol-binding and catalytic sites are highly conserved in qNor, and, thus, apparently important for nitric oxide reduction to nitrous oxide, but not in qNor-related or *nod* sequences (12, 15, 21, 22). The putative *nod* sequences recovered from our samples consistently had deviations in sites His328, Glu332, and Phe336, as reported by others (12, 16). Interestingly, 55 of our 88 (66%) sequences consistently had His328 substituted with threonine and Glu332 substituted with arginine at the quinol-binding site. This substitution pattern was consistent across all sites sampled and was most prevalent in the NC10 and aquifer clusters. Notably, His328 and Glu332 were replaced by threonine and arginine, respectively, in other reported *nod* sequences from agricultural and freshwater sites. In a study of alpine wetlands, 8 of 21 reported *nod* sequences contained these substitutions surrounding the quinol-binding site (16), and in a different study these substitutions were observed in 4 of 26 reported sequences (12). While this amino acid substitution pattern is present in oxygenic denitrifiers from a variety of environmental locations, it does not appear to be common in *nod* sequences in engineered systems (e.g., wastewater treatment plant reactors). Furthermore, His560 was consistently replaced by asparagine in *nod*, further suggesting an altered active site as supported by previous work (12, 15).

10.1128/msphere.00571-22.3 FIG S1 Sampling locations were chosen to include freshwater riverbank (a) and agricultural sites (b). Download FIG S1, TIF file, 12.4 MB. Copyright © 2023 Schmitz et al. 2023 Schmitz et al. https://creativecommons.org/licenses/by/4.0/ This content is distributed under the terms of the Creative Commons Attribution 4.0 International license.

### Distribution and abundance of *nod* genes in soils and sediments.

We expected that *nod* genes would occur in greater quantities at the farm sites compared to the freshwater sites due to the higher availability of nitrogen compared to the freshwater samples. We quantified the abundance of *nod* genes and bacterial 16S rRNA genes at our different sampling locations with quantitative PCR (qPCR). Comparing *nod* gene abundance to 16S rRNA gene abundance provides information about the respective amounts of oxygenic denitrifiers relative to total bacterial abundance and the relative niche space of oxygenic denitrifiers.

We found that *nod* genes were indeed more abundant in agricultural soils compared to freshwater sediments. The greatest *nod* abundances in reconnaissance samples occurred in the Farm Tile Drain soil (4.3 × 10^6^ copies/g soil) and Farm Bog soil (4.5 × 10^6^ copies/g soil) samples at the JCHPF (Table 1). The greatest relative abundance of *nod* genes, normalized to total 16S rRNA genes, was 0.045% in the Farm Tile Drain soil and 0.054% in the Farm Bog soil, while the lowest relative abundance of *nod* genes was detected from the Freshwater River Bank sediment sample (Table 1).

The 2 farm sites with the greatest absolute and relative *nod* abundances were located at the outlets of tile drains. We speculate that these sites had higher readily available nitrogen from accumulation in the tile drains compared to the freshwater sites. Additionally, we speculate these sites had greater methane availability compared to the freshwater sites which would support *nod*-harboring n-DAMO microbes. Further investigation of the physiochemical properties of these sites should be conducted to confirm these assumptions.

Overall, our findings are in accordance with reported environmental *nod* abundances (12, 16–18). Our speculation that nitrogen availability impacts *nod* abundance is further supported when comparing our findings with reported quantities of *nod* genes. The JCHPF samples were taken from an artisan farm with low nitrogen fertilizer inputs. The *nod* abundances reported in alpine wetland soil (16), where relatively low nitrogen concentrations were present, were lower than all our farm sites but similar to our freshwater *nod* magnitudes. The absolute and relative *nod* abundances from the agricultural soil applied with nitrogen fertilizer were greater than our reported values, as well as those from alpine wetland (16, 17). Additionally, it makes sense that the BTEX-impacted aquifer had one of the greatest absolute and relative *nod* abundances compared to our samples and others (12). The aquifer in that study contained BTEX, a suitable environment to harbor all 3 putative *nod* previously identified in the genomes of 2 different bacteria (DAMO_2434, DAMO_2437, and HdN1 *nod*). Even though our environmental survey aimed to detect all nod genes, the agricultural sites did not contain HdN1 *nod* as expected due to the lack of long chain aliphatic hydrocarbons in the sample sites. While previous reports have analyzed the influence of nitrogen availability on *nod*-harboring n-DAMO bacteria in the environment, the direct investigation of *nod* gene abundance versus nitrogen availability is limited. We expect that *nod* abundance will follow the same pattern as n-DAMO bacteria and with greater *nod* gene abundances associated with greater nitrogen availability. Our study highlights the need for future investigation of the influence of nitrogen availability and other physiochemical properties on *nod* gene abundance.

### Abundance of *nod* and total 16S rRNA genes in JCHPF core samples.

We expect that fully saturated zones at agricultural sites provide a suitable niche space for *nod*-harboring bacteria. In a previous study, greater abundance of n-DAMO biomarkers was observed in saturated lake sediments compared to variably saturated lake sediments (17) but *nod* was not explored. Further evidence to support this assertion revealed that *nod*-harboring n-DAMO bacteria occurred close to oxic/anoxic interfaces with high concentrations of available methane and nitrate (19, 23, 24).

**FIG 3:**
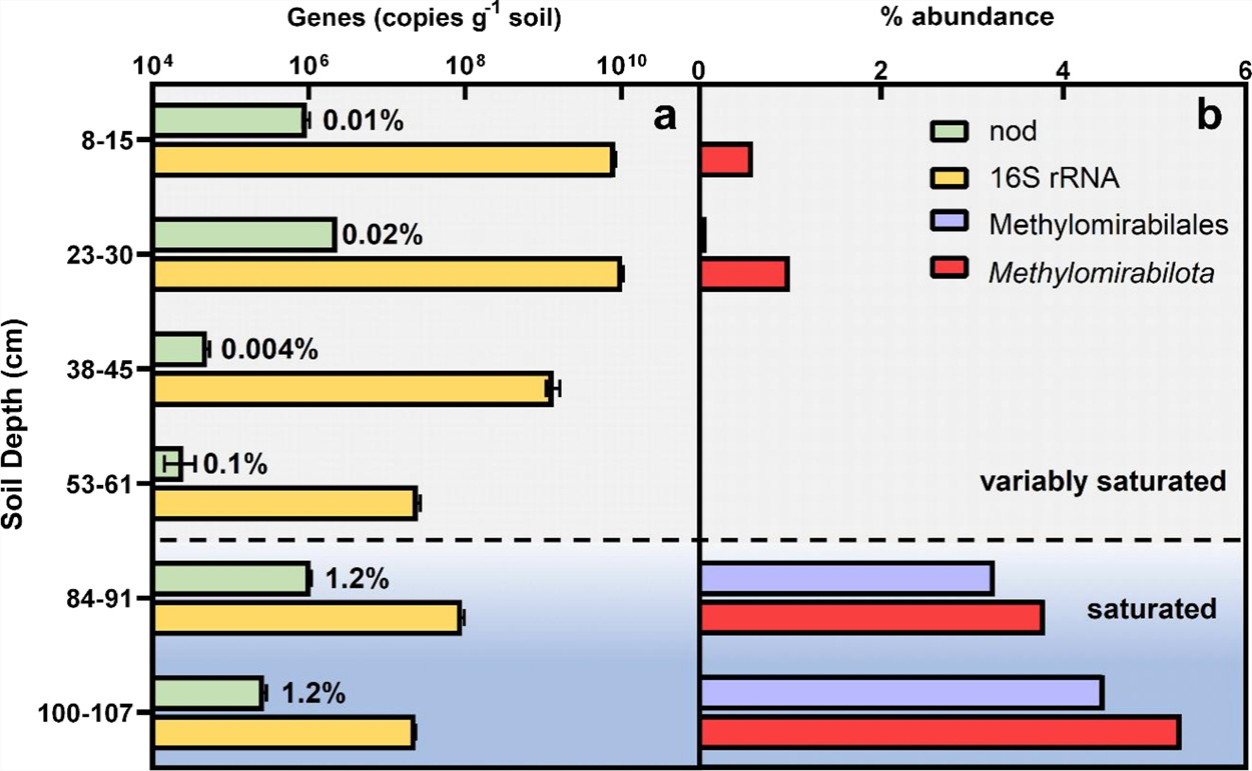
(a) Depth profile for 16S rRNA and *nod* gene copies per g soil, as determined by qPCR, for the farm grass waterway soil column. (b) Depth profile of relative 16S rRNA gene abundances for the phylum Methylomirabilota and the order *Methylomirabilales*, as determined by 16S rRNA gene sequencing, for the Farm grass waterway soil column. The approximate depth at which soil moisture conditions transition from variably saturated to fully saturated is indicated with a dashed line.

To investigate the relative abundance of oxygenic denitrifiers to soil saturation conditions, we compared the relative abundance of *nod* to 16S rRNA genes as a function of depth in a soil core collected from the JCHPF. The soil core ranged from variably saturated soil to fully saturated soil with depth (Fig. 3). The *nod* gene abundances were between 2.5 × 10^4^ and 2.3 × 10^6^ gene copies g soil^−1^. We showed that the variably saturated core sample had a *nod* to 16S rRNA gene ratio of 0.004% to 0.1%, and that the fully saturated core samples had a *nod* to 16S rRNA gene ratio of 1.2% (Fig. 3). The more than 10-fold increase in relative *nod* abundance supports our hypothesis that fully saturated soils provide a favorable niche for *nod*-harboring bacteria. Furthermore, these results suggest that *nod* genes potentially play a more important role in nitrogen cycling in fully saturated soils compared to variably saturated soils.

To further investigate the effect of soil saturation on oxygenic denitrification, we investigated the 16S rRNA gene composition of four sub-core samples. We found the relative abundance of 16S rRNA genes classified as belonging to the phylum *Methylomirabilota* to total 16S rRNA sequences increased from 0.6% and 1% in the top 2 soil sub-cores (8-15 cm and 23-30 cm) to 3.8% and 5.3% in the bottom 2 soil sub-cores (84-91 cm and 100-107 cm). The phylum *Methylomirabilota* is currently comprised of 2 orders, *Rokubacteriales* and *Methylomirabilales*, of which only *Methylomirabilales* are oxygenic dentrifiers (9, 25). Our analysis of the soil core demonstrated that the relative abundance of the order *Methylomirabilales* 16S rRNA genes to total 16S rRNA genes increased from 0.01% and 0.09% in the top 2 soil sub-cores to 3.25%, and 4.45% in the bottom 2 soil sub-cores.

The increased relative abundance of *Methylomirabilota* and *Methylomirabilales* indicates an increased abundance of oxygenic denitrifiers, specifically NC10 members. It should be noted that the relative abundance of general nod genes was less than that of *Methylomirabilales*, most likely due to different quantification methodologies (qPCR versus Illumina sequencing). The greatest relative abundance of *nod* genes in our study occurred in the fully saturated soil core near this interface, represented by the water table level. Our findings suggest that oxygenic denitrifiers could be sensitive to soil saturation and that fully saturated zones could be a more suitable niche space for oxygenic denitrifiers than variably saturated zones. Interestingly, while the abundance of NC10 members increased with soil saturation, the soil core phylogenetic diversity indicated that it was mostly comprised of members other than those with nod sequences in the NC10 cluster (Fig. 1). This highlights the need for future research for other oxygenic denitrifiers and supports the need for *nod*-targeted gene assays to cover the full spectrum of oxygenic denitrifiers.

Our findings suggest that greater soil saturation could enhance the growth of oxygenic denitrifiers and, subsequently, nitrite and nitrous oxide minimization. Current efforts to reclaim the nitrate and nitrous oxide mitigation capacity of the soil profile in Iowa include the installation of control structures at tile outlets so that farmers can elevate the water table after spring planting. For example, at the farm where the soil core was collected, there are 6 constructed weirs that control the water table, and, thus, the saturation of the sub-surface. Using these weirs to raise the water table could select for conditions that favor more oxygenic denitrifiers and their sustainable nitrate and nitrous oxide mitigation potential.

### Conclusions.

Agricultural soils, including previously unexplored environments, such as tile drains, contain diverse and abundant oxygenic denitrifiers as indicated by *nod* sequences. This study has expanded the known diversity of *nod* genes in the environment, especially from previously unexplored environments, such as tile drainage sites. Phylogenetic and residual substitution analysis confirmed that novel *nod* sequences were recovered from these sites, and expand the database of *nod* genes. Our work helps to further establish *nod* genes as a biomarker for oxygenic denitrification and highlights the need for directly investigating *nod* genes to recover the full spectrum of oxygenic denitrifiers. Furthermore, our reconnaissance of *nod* genes provided additional evidence for *nod* gene quantification in freshwater and agricultural soil and, to our knowledge, the first quantification of *nod* genes at tile drainage sites. There is a need for further identification and analysis of environmental *nod* sequences. Furthermore, through investigating the relationship between soil saturation, *nod* abundance, and *Methylomirabilota* abundance, we suggest that fully saturated zones are a more suitable niche for oxygenic denitrifiers compared to variably saturated zones. Further investigation of the influence of saturated conditions on oxygenic denitrifier abundance should be conducted.

## MATERIALS AND METHODS

### Reconnaissance surface samples.

Sampling locations were chosen to include agricultural sites and freshwater riverbank sites influenced by agricultural practices (Fig. S1). Furthermore, the specific site from which the sediments or soil were collected at each sampling location was based on assumptions about reducing conditions, saturation, and nitrogen availability. Three surface samples from the Johnson County Historic Poor Farm (JCHPF) were collected from a broccoli patch, the outlet of a tile drain, a drainage ditch, and at a bog. Three freshwater sediment samples were collected from 2 sites on Ralston Creek and one on the Iowa River. Sediment samples from each location were placed in sterile 50 mL centrifuge tubes, stored on ice, and immediately brought to the lab for analysis.

### Soil core.

To further explore the farm site and influence of soil saturation on *nod* genes, a soil core (6 cm diameter) was collected to a depth of 1.2 m in a depositional environment located at the base of a grass covered waterway using a hydraulic coring system. The soil was characterized at the time of sampling. The texture for the top 36 cm of the soil profile was classified as a silt loam. Soils from 36 to 120 cm were classified as a silty clay loam. The 122 cm soil core was processed by dividing into 7.6 cm sub-cores (total of 16), and then each sub-core was split in half vertically. Every other sub-core was homogenized, and 0.25 g of sediments were collected for DNA isolation.

### DNA extraction.

DNA was extracted from surface and core sediment samples immediately after collection using the DNeasy PowerSoil Pro Kit (Qiagen). The quantity of the DNA was determined using a Qubit 4 fluorometer and the Qubit dsDNA HS assay kit (Thermo Fisher Scientific). Isolated DNA was stored at −20°C until analysis.

### PCR and qPCR.

To screen for *nod* genes in the isolated DNA, PCRs were performed with primers *nod* 684Fv2 (5′-STAYACHCAYAACTGGCC-3′) and *nod* 1706Rv2 (5′-GGTGBYBTTCCTGTTCTTYRG-3′) targeting general *nod* genes (20). All PCRs were performed in 25 μL reactions containing 12.5 μL *Taq* Master Mix (Qiagen), 0.5 μM each primer, 1 μL template DNA, and balanced with nuclease-free water. PCR thermocycler conditions were 3 min at 96°C, followed by 30 cycles at 95°C (45s), 57°C (60s), 72°C (90s), followed by 5 min at 72°C. The presence and approximate size of PCR products were verified by electrophoresis using a 2% agarose gel.

After initial PCR screening, *nod* and 16S rRNA gene abundances in selected samples were quantified by qPCR using the *nod* 1446F (5′-GGCTTSGCRATCCAGTAGAAG-3′) and *nod* 1706Rv2 (5′-GGTGBYBTTCCTGTTCTTYRG-3′) and 16SU-f (5′-TCCTACGGGAGGCAGCA GT-3′) and 16SU-r (5′-GGACTACCAGGGTATCTAATCCTGTT-3′) primer sets, respectively. A synthetic *nod* gene fragment (gBlock; Integrated DNA Technologies), designed using *M. oxyfera* DAMO_2437 (FP565575.1), was used as a qPCR standard for *nod* genes (Table S1). The 16S rRNA gene from Paraburkholderia xenovorans LB400 (GenBank Acc. No. NR_074325) was used as a qPCR standard for total 16S rRNA genes. Standard DNA templates were quantified in triplicate, and samples were quantified in duplicate. All qPCRs were performed with an ABI QuantStudio 7 Flex real-time PCR system (Applied Biosystems) in 20 μL reactions containing 10 μL Power SYBR green PCR Master Mix (Invitrogen), variable primer and template concentrations, 100 ng/μL bovine serum albumin, and balanced with nuclease-free water (Table S2). The qPCR thermocycler conditions were: 10 min at 95°C, followed by 40 cycles at 95°C (15s), 60°C (60s), 72°C (60s), followed by 5 min at 72°C. To quantify the unknown sample gene abundances, a standard curve was constructed by creating serial dilutions of the template standards containing 30 to 3 × 10^7^ gene copies. After amplification, the targeted standard concentrations were plotted against the resulting Ct values, and a linear regression was performed to develop the slope and intercept of the standard curve. The unknown sample Ct values were then compared against the standard curve to determine their quantity. qPCR quality assurance and quality control procedures were performed in accordance with MIQE guidelines by analysis of no template control (NTC) (26). Inspection of the melt curves showed single peaks, indicating specific amplification of the genes of interest. Acceptable calibration curves had an R^2^ value of 0.99 or greater and a PCR efficiency between 90 and 110%. Gene abundance for each sample quantified by qPCR was normalized per g soil mass.

10.1128/msphere.00571-22.1 TABLE S1 Pertinent sequence information for qPCR calibration curves. Download Table S1, DOCX file, 0.01 MB. Copyright © 2023 Schmitz et al. 2023 Schmitz et al. https://creativecommons.org/licenses/by/4.0/ This content is distributed under the terms of the Creative Commons Attribution 4.0 International license.

10.1128/msphere.00571-22.2 TABLE S2 Pertinent qPCR parameters (primer concentration, template mass, linear range, PCR efficiency, and y-intercept of the qPCR standard curve). Download Table S2, DOCX file, 0.01 MB. Copyright © 2023 Schmitz et al. 2023 Schmitz et al. https://creativecommons.org/licenses/by/4.0/ This content is distributed under the terms of the Creative Commons Attribution 4.0 International license.

### Cloning and sequencing of *nod* PCR products.

The *nod* sequence diversity was further explored in samples that passed the initial *nod* PCR screening process. This was accomplished through cloning and sequencing of the PCR products. PCR products were first purified using a QIAquick PCR purification kit (Qiagen), and then inserted in the pCR 2.1-TOPO vector using the TOPO TA Cloning Kit (Invitrogen). The cloning reaction was incubated at room temperature for 1 h. Cloned vector was transformed into One Shot TOP10 chemically competent E. coli cells (Invitrogen). Transformed cells were plated on LB agar containing 50 mg/L kanamycin and 1.6 mg/plate X-gal and incubated overnight at 37°C. Individual clones were grown in LB plus 50 mg/L kanamycin and incubated overnight at 37°C. The PureLink Quick Plasmid Miniprep Kit (Invitrogen) was used to purify plasmid DNA from overnight cultures. Clones were Sanger sequenced at the Iowa Institute of Human Genetics Genomic Division using vector primers M13F (5′-TGTAAAACGACGGCCAGT-3′) and M13R (5′-CAGGAAACAGCTATGAC-3′).

### 16S rRNA sequencing.

Sequencing of 16S rRNA gene amplicons from selected soil sub-cores (8-15 cm, 23-30 cm, 84-91 cm, and 100-107 cm) ranging across variably saturated to fully saturated conditions was performed at MR DNA (Shallowwater, TX) using Illumina Miseq and the 515F (5′-GTGCCAGCMGCCGCGGTAA-3′) and 806R (5′-GGACTACHVGGGTWTCTAAT-3′) primer set (12). Sequences were further analyzed using the DADA2 pipeline and the Phyloseq R package for analysis (27, 28). Briefly, samples were split into individual per-sample fastq files, primers were removed, paired sequences were joined, and short or ambigous sequences were removed. The remaining sequences were quality filtered, assigned to an amplicon sequence variant, which was then assigned taxonomy from the Silva Project’s version 138.1 database (29).

### Phylogenetic analysis.

The sequences obtained were translated to amino acid sequences and aligned with reference sequences retrieved from NCBI using the ClustalW algorithm with default settings in MEGAX (30–32). Then, a phylogenetic tree was constructed using the neighbor-joining method with the robustness of the tree topology tested by bootstrap analysis (500 replicates).

### Residual substitution analysis.

Multiple amino acid sequence alignments of selected qNor and qNor-related enzymes and inferred *nod* amino acid sequences retrieved in this study were performed in MEGAX with ClustalW to identify the expected quinol-binding and catalytic sites, as described previously (30–32).

10.1128/msphere.00571-22.4 FIG S2 Unabridged multiple alignment around the expected quinol-binding and catalytic sites of selected qNor and qNor-related amino acid sequences with selected inferred *nod* amino acid sequences obtained in this study. Conserved quinol-binding and catalytic site residues found in qNor sequences are highlighted in red, whereas substitutions to those conserved residues in putative *nod* sequences are shown in green. The alignment was created in MEGAX with ClustalW using default parameters (32). Download FIG S2, TIF file, 5.3 MB. Copyright © 2023 Schmitz et al. 2023 Schmitz et al. https://creativecommons.org/licenses/by/4.0/ This content is distributed under the terms of the Creative Commons Attribution 4.0 International license.

### Data availability.

The high-throughput partial 16S rRNA gene sequencing data generated in this study were deposited in the GenBank Sequence Read Archive under BioProject number PRJNA879082. The *nod* genes retrieved from soil and sediment samples are deposited under GenBank accession numbers OP679926-OP680013.
