# Supplementary figures and images for: Reconnaissance of Oxygenic Denitrifiers in Agriculturally Impacted Soils
Source: mSphere. 2023 Apr 5;8(3):e00571-22. doi: 10.1128/msphere.00571-22 (PMC10286720; doi:10.1128/msphere.00571-22)

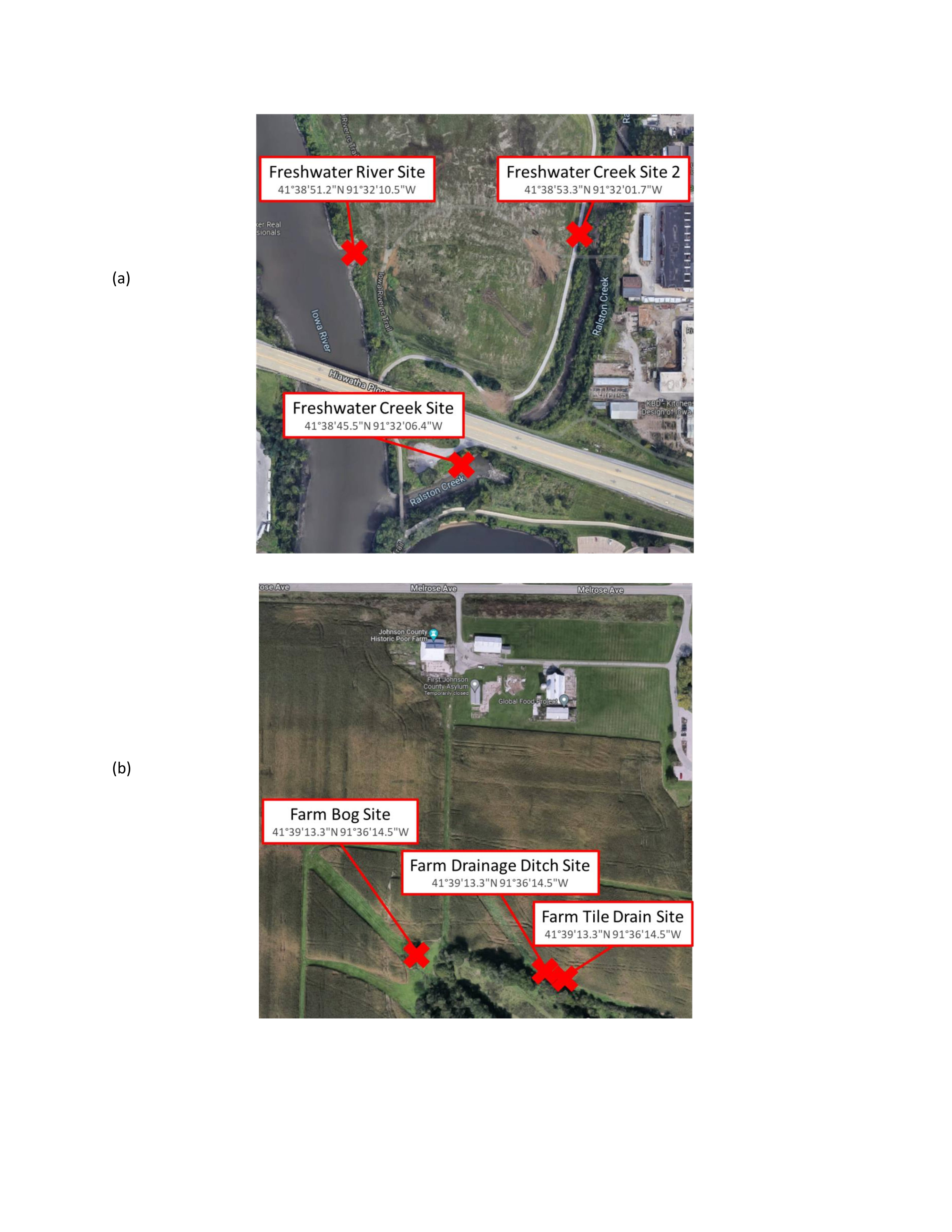

Supplement: FIG S1 [file msphere.00571-22-s0003.tif]
