# Supplementary material for: Reconnaissance of Oxygenic Denitrifiers in Agriculturally Impacted Soils
Source: mSphere. 2023 Apr 5;8(3):e00571-22. doi: 10.1128/msphere.00571-22 (PMC10286720; doi:10.1128/msphere.00571-22)
Supplement: TABLE S2 [file msphere.00571-22-s0002.docx]

| Target gene | bacterial 16S rRNA gene | nod (1446F-1706Rv2) |
| --- | --- | --- |
| primer conc, μM | 0.1 | 0.5 |
| template | 2 ul DNA | 2 ul DNA |
| qPCR linear range, gene copies/reaction | 30 - 3 × 10^7^ | 30 - 3 × 10^7^ |
| qPCR efficiency | 90-110% | 90-110% |
| Y-intercept | 36.6-37.3 | 38.3-39 |
| R^2^ | >0.97 | >0.98 |
